# Blocking Effect of Demethylzeylasteral on the Interaction between Human ACE2 Protein and SARS-CoV-2 RBD Protein Discovered Using SPR Technology

**DOI:** 10.3390/molecules26010057

**Published:** 2020-12-24

**Authors:** Zhi-Ling Zhu, Xiao-Dan Qiu, Shuo Wu, Yi-Tong Liu, Ting Zhao, Zhong-Hao Sun, Zhuo-Rong Li, Guang-Zhi Shan

**Affiliations:** Institute of Medicinal Biotechnology, Chinese Academy of Medical Sciences and Peking Union Medical College, Beijing 100730, China; zhiling.zhu@live.com (Z.-L.Z.); xdqsdu@163.com (X.-D.Q.); wushuoimb@126.com (S.W.); LiuYT8210@163.com (Y.-T.L.); zhaoting@imb.pumc.edu.cn (T.Z.); sunzhonghao@imb.pumc.edu.cn (Z.-H.S.)

**Keywords:** SARS-CoV-2, COVID-19, surface plasmon resonance, protein–protein interaction inhibitors, ACE2, receptor binding domain

## Abstract

The novel coronavirus disease (2019-nCoV) has been affecting global health since the end of 2019, and there is no sign that the epidemic is abating. Targeting the interaction between the severe acute respiratory syndrome coronavirus 2 (SARS-CoV-2) spike protein and the human angiotensin-converting enzyme 2 (ACE2) receptor is a promising therapeutic strategy. In this study, surface plasmon resonance (SPR) was used as the primary method to screen a library of 960 compounds. A compound 02B05 (demethylzeylasteral, CAS number: 107316-88-1) that had high affinities for S-RBD and ACE2 was discovered, and binding affinities (K_D_, μM) of 02B05-ACE2 and 02B05-S-RBD were 1.736 and 1.039 μM, respectively. The results of a competition experiment showed that 02B05 could effectively block the binding of S-RBD to ACE2 protein. Furthermore, pseudovirus infection assay revealed that 02B05 could inhibit entry of SARS-CoV-2 pseudovirus into 293T cells to a certain extent at nontoxic concentration. The compoundobtained in this study serve as references for the design of drugs which have potential in the treatment of COVID-19 and can thus accelerate the process of developing effective drugs to treat SARS-CoV-2 infections.

## 1. Introduction

The emergence of severe acute respiratory syndrome coronavirus 2 (SARS-CoV-2) has caused a global outbreak of coronavirus disease 2019 (COVID-19) [1,2]. SARS-CoV-2 infection can cause symptoms such as fever, dry cough and dyspnea. Unlike SARS, upper respiratory symptoms and intestinal presentations of COVID-19 are not common [1]. To date, the number of COVID-19 cases confirmed worldwide has exceeded 49.1 million, with a continuing upward trend [3].

Through the concerted efforts and dedication of global scientific researchers, the biological characteristics of SARS-CoV-2 have been briefly elucidated [4]. The S protein of SARS-CoV-2 mainly mediates binding with the membrane receptor on the host cells (angiotensin-converting enzyme 2; ACE2) and facilitates viral entry into host cells [5,6,7,8,9,10]. The receptor binding domain (RBD) of the S protein is directly involved in the recognition of host receptors. Amino acid variations in this region cause changes in the species’ tropism and infection characteristics [11,12].

Considering the severe pandemic situation, there is an urgent need to search for effective treatment strategies for COVID-19. A vaccine against COVID-19 is among the most promising methods to end the pandemic. Antiviral drug development is another effective countermeasure. Repurposing of drugs or drug leads, as well as the application of computational methods and high-throughput screening, are the main strategies used in the search for effective drugs against SARS-CoV-2 [13]. Among them, the repurposing of drugs is definitely the most efficient [13]. The COVID-19 pandemic has prompted worldwide interest in the repurposing of drugs such as remdesivir [14,15]. Currently, SARS-CoV-2 inhibitors are being screened actively, and various promising compounds have been obtained [16,17,18,19]. Dai et al. designed and synthesized two lead compounds targeting the SARS-CoV-2 main protease by structure-based design [20]. The results showed that both compounds possessed good pharmacokinetic properties in vivo, and one of the compounds also exhibited low toxicity. These two compounds are promising candidates for further clinical study. Entry into the host cell is the first step of virus infection, and therefore, blocking virus entry into the host cell is an effective strategy in developing drugs against COVID-19 [21,22]. Recently published virtual screening studies targeting S-RBD and ACE2 protein suggest that natural products such as glycyrrhizic acid, emodin and hesperidin could inhibit the binding of SARS-CoV-2 to ACE2 [23]. However, the antiviral activity and efficiency of most compounds is still unclear.

S-RBD and ACE2 proteins recognize protein–protein interactions (PPIs) through their interaction surfaces. This poses a challenge while using traditional screening methods for drugs targeting these interactions. Surface plasmon resonance (SPR) is a versatile biophysical detection technology developed in the 1990s [24,25]. It is a label-free and real-time molecular interaction analysis technology [26]. It can be used to characterize various binding interactions, which include not only highly potent PPIs but also weak fragment or low molecular weight (LMW) ligand–protein interactions. When used for screening, SPR allows simultaneous acquisition of detailed kinetic characterization of drugs in real time. Additionally, it is a powerful tool for discovering PPI inhibitors [27]. Compared with traditional inhibitor screening based on enzyme activity, SPR technology is not dependent on the enzyme reaction process. This screening technology is aimed at “full site” screening of target proteins.

In this study, SPR technology was used to screen a library of 960 compounds, with S-RBD and ACE2 proteins as the targets. All compounds have been approved for clinical phase, classified according to clinical phase. Of the hits from this screen, demethylzeylasteral was found as the most promising compound to advance. It could bind to ACE2 and S-RBD with K_D_ values of 1.736 and 1.039 μM, respectively. Furthermore, the results of pseudovirus entry assay showed that the compound also had an effect on the entry of SARS-CoV-2 into host cells.

## 2. Results and Discussion

### 2.1. Surface Plasmon Resonance Assay Development and Protein–Protein Interaction Testing

**Figure 1:**
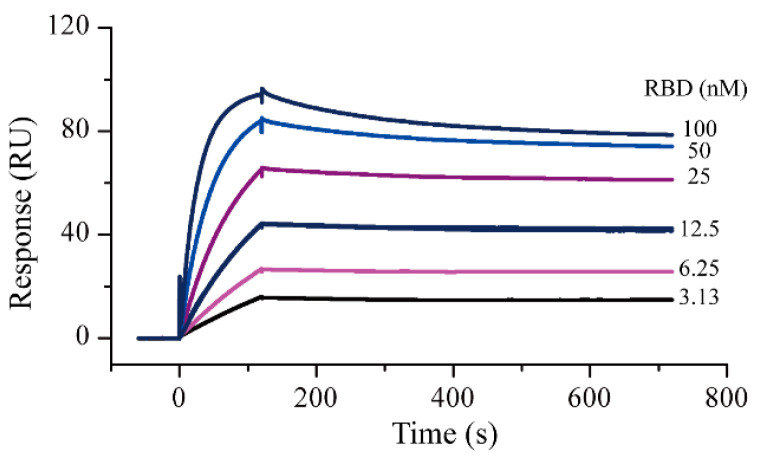
Surface plasmon resonance sensorgrams for S-RBD-mFc interaction with captured ACE2-His. Increasing concentrations of S-RBD were injected over the surface. Contact time and dissociation time was kept at 120 and 500 s, respectively. Data were analyzed with Biacore S200 evaluation software (v 1.0) and automatically fitted to the 1:1 binding model.

Amine coupling and capture methods were used as the protein immobilization methods in the SPR experiments. Compared with amine coupling, the capture method caused negligible changes in the 3D structure of the proteins. In this study, the ACE2-His protein was first captured as a ligand and S-RBD-mFc was used as an analyte to evaluate the activity of the two proteins. The running buffer solution was selected according to a recently reported study [7]. Results showed that the ACE2 capture quantity in different cycles was relatively stable; the response was about 143 RU and the deviation did not exceed 2 RU. Using this interaction method, the ACE2-S-RBD interactions were measured (Figure 1). The measured S-RBD maximum response signal was 94.2 RU, which did not differ significantly from the theoretical Rmax of 87.7 RU. These results demonstrated that the activity of both the proteins remained good in the running buffer. After 1:1 data fitting, the affinity constant obtained was 0.37 nM, which was similar to the value (0.562 nM, determined by Octet RED system) specified in the instructions provided by the protein supplier. After completing the protein activity assessment, SPR screening models for S-RBD and ACE2 were established with the goals of reducing the protein consumption as much as possible and improving the screening efficiency. By referring to the protocol provided by the instrument manufacturer, the amine coupling method was used for protein immobilization. Based on the molecular weights of the compound to be screened and the target protein, the signal response for ACE2 protein immobilization had to be greater than 8500 RUs (greater than 5000 RUs for S-RBD protein) for subsequent screening. Finally, the response of S-RBD protein and ACE2 were both approximately 12,000 RU (Figures S1 and S2 in Supplementary Materials).

### 2.2. Screening and Affinity Analysis

**Figure 2:**
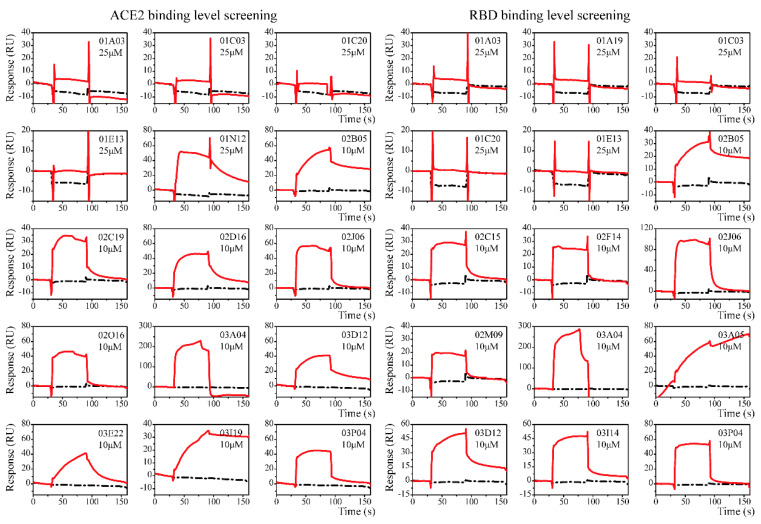
The sensorgrams of the Binding Level Screen hits. Binding curves of immobilized ACE2 (**left**) and S-RBD (**right**). Data are shown as red lines, and the blank control data are shown in black. Response with molecular weight adjustment was the selection theory. Top five compounds on each plate (three plates in total) were chosen as the hits.

Clean Screen was carried out to eliminate compounds that showed non-specific or residual binding to the sensor chip matrix, which could have an effect on the data quality of subsequent injections. Based on the difference in the solubilities of the compounds, some compounds were formulated at a concentration of 25 μM, while others were formulated at a concentration of 10 μM. Thirteen compounds were excluded due to their high viscosity. Compounds that passed the Clean Screen were then examined through the Binding Level Screen to identify compounds that could bind to the targets of interest. Since the targets involved in this study are relatively novel and there is a lack of positive control compounds, the compounds with the top five signal response in each plate were selected for affinity determination (Figure 2).

**Figure 3:**
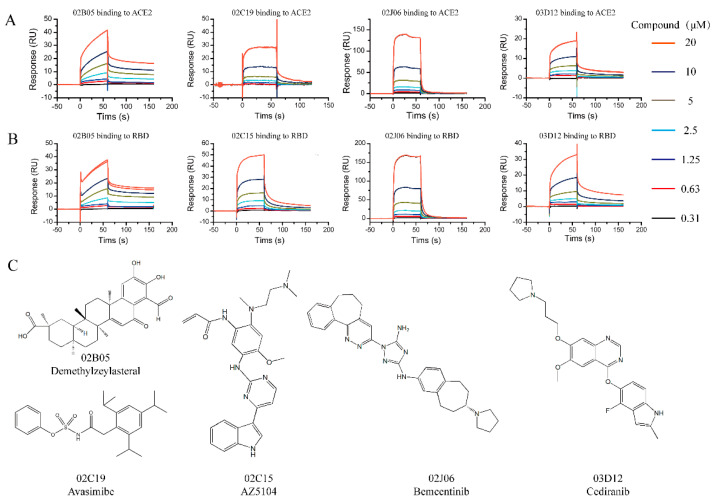
The sensorgrams and structures of compounds identified from the affinity screening. (**A**) Sensorgrams of compounds identified from the affinity screening bound to ACE2; (**B**) sensorgrams of compounds identified from the affinity screening bound to S-RBD. Increasing concentration of compounds were injected over the chip surface. The contact time was 60 s and dissociation time was 120 s. (**C**) Chemical structures of compounds discovered during SPR affinity screening.

Twenty-one compounds that bound to ACE2 or S-RBD in the Binding Level Screen were subsequently assayed in a dose–response experiment to determine the binding affinity. Different concentrations of the compounds (0.3125~20 μM) were flowed over the surface of the chip immobilized with ACE2 or S-RBD protein. There were five compounds (02B05, 02C15, 02J06 and 03D12) binding to S-RBD and four compounds (02B05, 02C19, 02J06 and 03D12) binding to ACE2, with K_D_ values < 100 μM (Figure 3).

Residence time is a potential indicator used in drug discovery. Generally, a long residence time of a compound is closely associated with the magnitude and duration of its efficacy [28]. Prolonging the residence time is beneficial in improving the success rate of drug development [28]. Using a kinetic fitting model, the kinetic constants of several compounds and the two targets were measured. The detailed data are shown in the Table 1. The sensorgrams and drug structures are shown in Figure 3 and Figure S3 in Supplementary Materials. The time required for the drug to associate with the target is mainly reflected by the association rate constant (*k_on_*), while the duration of target occupancy is primarily governed by the dissociation rate constant (*k_off_*). The dissociation rate constant of the compound 02B05 was the lowest, indicating that it had the longest residence time. These results indicated that the compound, 02B05, is expected to be a potential S-RBD/ACE2 binding inhibitor.

### 2.3. Validity of Interaction by Isothermal Titration Calorimetry (ITC)

**Figure 4:**
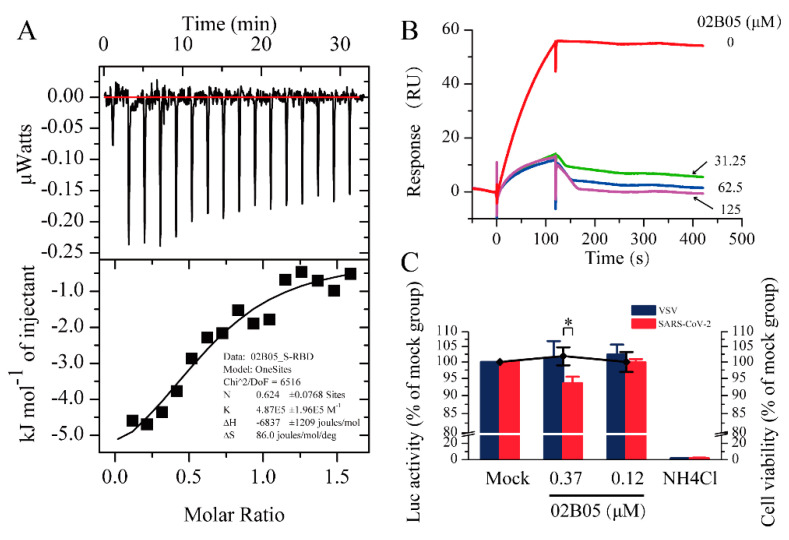
The results of Isothermal Titration Calorimetry (ITC), surface plasmon resonance (SPR) competition experiments and pseudovirus entry assay. (**A**) Thermodynamic analysis of the binding of 02B05 to S-RBD-His was carried out at 25 °C on a MicroCal ITC 200 instrument. (**B**) Competitive inhibition of 02B05 on ACE2-S-RBD. S-RBD (20 nM) was incubated with compounds at various concentrations and then run over ACE2. (**C**) The effect of compound 02B05 on the transduction of pseudotyped VSV or SARS-CoV-2 virus in 293T-hACE2 cells. The 293T-hACE2 cells seeded in 96-well plates were mock treated or treated with the indicated concentrations of compound 02B05. Cell viability was determined using a PrestoBlue cell viability reagent. The 293T-hACE2 cells were also infected with VSV or SARS-CoV-2 pseudovirus in the presence of the indicated concentrations of compound 02B05 or 20 mM of ammonium chloride. At 24 h post infection, luciferase activity in the cell lysates was determined. Relative transduction efficiency was characterized using luciferase (Luc) activity normalized to that of mock-treated cells and expressed as the mean ± standard deviation of two independent experiments in triplicates. * *p* < 0.05. The left and right Y-axis of the graphs represent mean % Luc activity and cell viability, respectively.

The SPR results showed that the affinity constant (K_D_) between *S*-RBD and 02B05 was the minimum. To confirm the results obtained by SPR, an ITC experiment was conducted to determine the affinity of 02B05-S-RBD. Considering the relatively large protein consumption of the ITC experiment, the S-RBD-mFc used in SPR experiment was replaced with S-RBD-His protein expressed by collaborative laboratory. The affinity measured by ITC was 2.09 μM and was close to that detected by SPR, within experimental error (Figure 4A). This suggested that compound 02B05 could bind to S-RBD-His in the running buffer solution. This result further confirmed the reliability and accuracy of the SPR screening method.

### 2.4. Surface Plasmon Resonance-Based Competition Assay

The competition experiment was conducted to investigate the blocking effect of compounds on the binding of S-RBD to ACE2. Six promising compounds in the Table 1 obtained in the SPR screening were selected for this experiment. The assay solution of 20 nM S-RBD, containing compounds from 0 to 125 μM, was serially flowed over the surface of ACE2 protein, and then the signal responses were observed. The relationships between response and compound concentrations are shown in Figure 4B and Figure S4 in Supplementary Materials. Among the compounds, 02B05 showed the strongest blocking effect (Figure 4B), followed by 03D12. The responses were significantly reduced by 02B05 and 03D12 in a dose-dependent manner, while the competitive inhibition of other compounds (125 μM) was not obvious. The data suggested that 02B05 and 03D12 was effective in blocking ACE2-S-RBD interaction, and were included in the later evaluation.

### 2.5. Pseudovirus Entry Assay

Since SARS-CoV-2 is a highly infectious and pathogenic virus, conducting research on it can be difficult and dangerous. To address this issue, a pseudovirus can be used as a safe and effective experimental tool since it has high security, wide host tropism and strong stability [29]. To investigate whether compound 02B05 and 03D12 inhibited virus entry into host cells, 293T-hACE2 cells were infected with luciferase reporter virus pseudotyped with SARS-CoV-2 spike envelope protein or VSV G protein, in the presence of compound 02B05 or 03D12. Firstly, the cytotoxicity of 02B05 and 03D12 was evaluated. As shown in Figure S5 and Figure 4C, the CC_50_ of 02B05 was 7.67 ± 0.79 μM and it had no significant effect on the activity of 293T-hACE2 cells when the concentration was no more than 1.1 μM, as determined by the Cell Counting Kit (CCK) and PrestoBlue cell viability reagent. However, the cell morphology was affected at this concentration. At 0.37 μM, no changes were observed in the cell viability and cell morphology. As for 03D12, its cytotoxicity was not obvious even the concentration was as high as 3.3 μM (Figure S6 in Supplementary Materials). The pseudovirus entry assay was then carried out under nontoxic concentration. As shown in Figure 4C, 0.37 μM of compound 02B05 inhibited the transduction of approximately 7% SARS-CoV-2 pseudovirus but had no effect on the transduction of VSV pseudovirus. In contrast, the positive control compound ammonium chloride almost abolished the infection of both the pseudoviruses (Figure 4C). Meanwhile, 03D12 did not exhibit significant inhibitory activity at any concentration (Figure S6). These results reflected that the ability of the SARS-CoV-2 spike pseudotyped virus to enter 293-hACE2 cells was reduced after treatment with 02B05. Although the compound had low inhibitory activity against SARS-CoV-2 pseudovirus, it can still provide a good starting point for drug design against SARS-CoV-2 infection.

### 2.6. Virtual Docking

**Figure 5:**
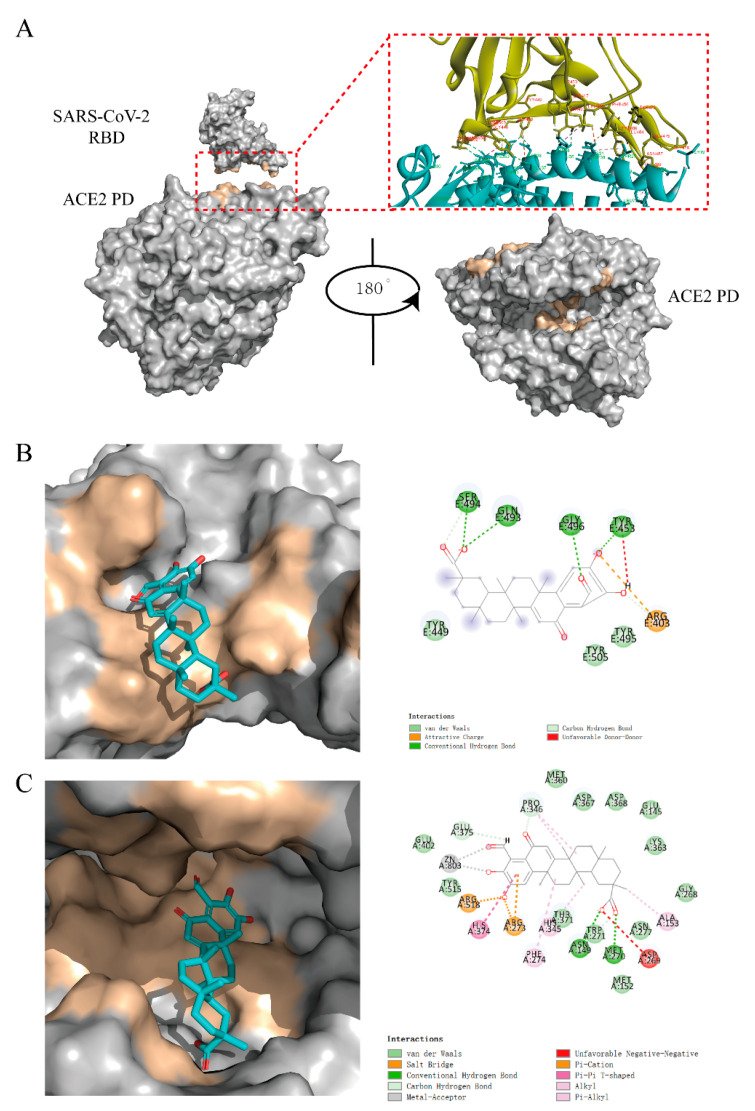
The results of docking. (**A**) Structure of the S-RBD-ACE2 protein–protein interaction (PPI) surface. The S-RBD/ACE2 is shown as a gray molecular surface (PDB ID: 6M0J), with residues that bind to ACE2/RBD colored yellow. The protease domain of ACE2 is shown as a gray molecular surface (PDB ID: 1R4L), with residues that bind to the S-RBD and substrate colored yellow. ACE2 PD (Right) was obtained by rotating ACE2 PD (Left) 180° to the right. (**B**) The interaction between S-RBD and 02B05 (left) and the key amino acids responsible for the interaction (right). (**C**) The interaction between 02B05 and the ACE2 enzyme activity site (left) and the key amino acids responsible for the interaction (right).

The binding region and the key amino acids of S-RBD and ACE2 were defined according to the published literatures and PDB database (Figure 5A). Using the binding area of the two proteins and the ACE2 enzyme active sites as the docking centers, the CDOCKER module of Discovery Studio 3.0 was used to virtually dock 02B05.

Compounds 02B05 could dock into the ACE2 binding area of S-RBD protein (Figure 5B). It formed hydrogen bonds with Tyr453, Gln493, Ser494 and Gly496 and van der Waals interactions with Tyr449 and Tyr505. Using the S-RBD binding area of the ACE2 protein on the surface as the docking center, the compound did not show good docking. Combined with the results of SPR, it is speculated that the compounds might bind to other sites of ACE2. Taking the ACE2 enzyme active sites as the docking region, the virtual docking results showed that the compound could bind well to it (Figure 5C).

## 3. Conclusions

The COVID-19 outbreak has caused huge losses in global health and the economy. The research and design of anti-SARS-CoV-2 drugs are of great significance. In this study, SPR was used as a primary screening method to obtain kinetics and affinity information simultaneously. This could avoid missing compounds with good kinetic characteristics but relative lower response signals. The compound, 02B05 (demethylzeylasteral), had a high affinity for the target protein and could affect the binding of the S-RBD to ACE2 according to the results of a competition experiment. The results of pseudovirus entry assay revealed that compound 02B05 could slightly prevent SARS-CoV-2 pseudovirus from infecting host cells at nontoxic concentration (0.37 μM). However, it also showed certain cytotoxicity (CC_50_ = 7.67 ± 0.79 μM). This implies that further structural optimization and modification of 02B05 are needed to reduce its toxicity and improve activity. Further virtual docking results showed that compounds 02B05 could bind to the ACE2 binding sites of S-RBD through hydrogen bonding and van der Waals forces. It could also bind to the ACE2 enzyme active sites. Taken together, the compound 02B05 obtained in this study could provide clues for the design of drug-like molecules with higher selectivity and better inhibitory activity against SARS-CoV-2 and form the basis of further structure optimization.

## 4. Materials and Methods

### 4.1. Chemicals and Reagents

The library, comprising 960 clinically available compounds, was kindly provided by Targetmol (Wellesley, MA, USA). The SARS–CoV-2 spike S-RBD protein (mFc tag, cat no: 40592-V05H, 51.5 kDa)and human ACE2 protein (His tag, cat no:10108-H08H, 85.1kDa ) were purchased from Sino Biological (Beijing, China), and the SARS–CoV-2 spike S-RBD protein with His tag (26.6 kDa) was kindly provided by the Institute of Materia Medica, Chinese Academy of Medical Sciences (Beijing, China). HBS-N buffer, an amine coupling kit, NTA reagent kit, acetate buffer, CM5 chips, and NTA chips were obtained from Cytiva (Washington, DC, USA). Dimethyl sulfoxide (DMSO), Tween 20 and sodium hydroxide were obtained from Sigma-Aldrich (Darmstadt, Germany).

The 293T-derived cell line, expressing human ACE2 (293T-hACE2), was purchased from Delivectory Biosciences Inc. (Beijing, China), and maintained in Dulbecco’s Modified Eagle Medium (DMEM; Invitrogen, Carlsbad, CA, USA), supplemented with 10% fetal bovine serum (FBS; Invitrogen, Carlsbad, CA, USA ) and antibiotics (100 U/mL penicillin and 100 mg/mL streptomycin), at 37 °C in a 5% CO2 incubator. PrestoBlue cell viability reagent was obtained from Invitrogen (Carlsbad, CA, USA). The vesicular stomatitis virus (VSV) and SARS–CoV-2 pseudotyped viral particles were obtained from Delivectory Biosciences Inc. (Beijing, China). Cell lysis buffer and luciferase substrate were purchased from Promega (Madison, WI, USA).

### 4.2. Protein–Protein Interaction Testing

The PPI of the ACE2 and S-RBD proteins was determined by their binding ability on an NTA chip, using a Biacore S200 instrument (Washington, DC, USA). An HBS-T+ buffer (10 mM HEPES, 150 mM NaCl, 0.05% Tween 20, pH 8.0, 0.22 μm filtered) was used as a running buffer. ACE2-His was captured as the ligand on the chip, at a concentration of 10 μg/mL. The capture time was 100 s. S-RBD-mFc was used as the analyte and was diluted in running buffer, with concentrations ranging from 3.15 to 100 nM. The buffer was allowed to flow over the captured ACE2-His, and the obtained response units (RUs) were recorded. The flow rate was set at 30 μL/min, with 120 s for binding and 500 s for dissociation. Next, the sensor chip surface was regenerated with 350 mM EDTA for 60 s.

For screening, the S-RBD-mFc protein and ACE2-His protein were immobilized on a CM5 sensor chip by amide coupling chemistry. The carboxylic acid groups were first activated by placing them in a mixture of N-Ethyl-N’-(3-dimethylaminopropyl) carbodiimide and N-hydroxysuccinimide. Then, S-RBD-mFc (20 μg/mL) and ACE2-His (16 μg/mL) proteins, dissolved in sodium acetate solution (pH 5.0 and 4.5, respectively) were immobilized on flow cell-2 (Fc2) and flow cell-4 (Fc4) of the CM5 chip, respectively. Finally, the chip was blocked with ethanolamine. To reduce non-specific interactions, the cell immobilized without protein was used as a reference.

### 4.3. Screening and Kinetic Analysis

The overall procedure for LMW affinity screening was divided into three general steps: Clean Screen, Binding Level Screen, and Affinity Screen [30]. All experiments were performed with Biacore T200 (Washington, DC, USA) or S200 instrument, at a flow rate of 30 μL/min. The data were analyzed using the Biacore T200/S200 Evaluation software. An HBS-T+ buffer (10 mM HEPES, 150 mM NaCl, 0.05% Tween 20, 5% DMSO, pH 8.0, 0.22 μm filtered) was used as the running buffer. Compounds were first diluted to 100 μM, which was 4- or 10-fold of the desired final concentration (25 or 10 μM), in running buffer with 5% DMSO. No regeneration operation was performed during the screening process. For the Clean Screen, compounds were injected at 25 μM or 10 μM, based on the differences in solubilities, using the CM5 sensor chip without preparation. The data were plotted as the baseline difference (RU) vs. cycle number. An alternative method is to generate a user-defined method using the early stability point (relative response) instead of the baseline difference [30].

For the Binding Level Screen, the chip was prepared using the amine coupling method, as described in Section 4.2. Compounds were screened at 25 or 10 μM on a surface of >5000 RU of S-RBD-mFc (Fc2) and > 8500 RU of ACE2-His (Fc4), with a 60 s contact time and a 60 s dissociation time. Running buffer was chosen as the negative control during screening. The solvent correction sample (eight point) that includes the 5% DMSO used in the assay was prepared according to the GE Healthcare Laboratory Guideline [31]. For data analysis, solvent correction was applied and the compound that lay outside the range was not corrected and noted. Adjustment for blank subtraction, molecular weight and controls were applied.

Binding Level Screen hits were then progressed into the Affinity Screen, with a two-fold concentration series from 0.3125 to 20 μM in running buffer. The contact time was 60 s and dissociation time was 120 s. Dose–response data were collected in the traditional multicycle format. The data were automatically fitted to the 1:1 binding model for both kinetics and steady-state affinity.

### 4.4. Interactions Determined by Isothermal Titration Calorimetry (ITC)

ITC was performed using a MicroCal ITC200 instrument (Malvern, UK). To ensure that the buffers matched, the solution of S-RBD-His protein and compound 02B05 were dissolved in the same buffer (10 mM HEPES, 150 mM NaCl, 0.05% Tween 20, 5% DMSO, pH 8.0). The titration experiments were performed in a sample cell (300 μL) containing 15 μM S-RBD-His protein and a syringe (40 μL) containing 100 μM compound. The reference power was set to 8 μcal/s, using a syringe stirring speed of 800 rpm. The thermal equilibration step at 25 °C was followed by an initial 60 s delay step. An initial injection of 0.2 μL was followed by 15 × 2 μL injections. The blank experiment was carried out under the identical operation conditions by directly injecting compound solution into the buffer. The data were fitted to a 1:1 binding isotherm.

### 4.5. Surface Plasmon Resonance-Based Competition Assay

The inhibitory effects of the hits were measured, as previously described in Section 4.2, with an NTA chip. The running buffer used was in accordance with the Affinity Screen buffer explained above (Section 4.3). The ACE2-His capture concentration was altered to 5 μg/mL and capture time remained the same. The assay solution of 20 nM S-RBD was prepared by dissolving S-RBD protein in the running buffer, containing compounds in the concentration range of 0 to 125 μM. The assay solution was injected over ACE2-His, with a time duration of 120 s for binding and 500 s for dissociation. Before the analysis, the S-RBD was incubated with compounds for 1 h at 25 °C.

### 4.6. Pseudotyped Lentiviral Particle Entry into Human Cells and the Luciferase Assay

First, cytotoxicity of 02B05 and 03D12 in 293T-hACE2 cells was assayed using PrestoBlue cell viability reagent. Briefly, the cells were seeded into 96-well plates and treated with different concentrations of compounds (a three-fold concentration series from 0.12 to 30 μM for 02B05 and 0.12 to 3.3 μM for 03D12). After incubation at 37 °C for 24 h, 10 μL PrestoBlue (10×) was added to each well and mixed well with the medium. After incubation at 37 °C for another 1 h, the fluorescent signals were read at an excitation wavelength of 560 nm and an emission wavelength of 590 nm, using the EnSpire multimode plate reader (Perkin Elmer, Waltham, MA, USA).

The 293T-hACE2 cells, seeded in white wall and clear bottom 96-well plates, were infected with pseudotyped VSV or SARS-CoV-2 viruses, in the absence or presence of the compound 02B05 and 03D12. Ammonium chloride was selected as the positive control. At 24 h post infection, the cells were lysed by incubating with cell lysis buffer (20 μL/well) for 15 min, followed by the addition of 50 μL/well of luciferase substrate. The firefly luciferase activities were measured by luminometry in a PerkinElmer EnSpire instrument. Entry efficiency was characterized using luciferase activity.

### 4.7. Docking

Based on the promising results obtained in the competition assay, compounds were virtually screened to validate the results of SPR. The crystal structure of the SARS-CoV-2 S-RBD-human ACE2 protein (protein data bank [PDB] ID: 6M0J) [5] and the crystal structure of the inhibitor-human ACE2 (PDB ID: 1R4L) [32] have been published in the PDB. Subsequently, the compounds were screened based on the SARS-CoV-2 S-RBD, the S-RBD binding sites of human ACE2 (saved from 6M0J), and human ACE2 enzyme active sites (obtained from 1R4L) using Discovery Studio 3.0, respectively. All above docking proteins were modified by deleting “Water” in the “Hierarchy view” to remove water molecules. Furthermore, the function of “Clean Protein” in Discovery Studio 3.0 was used to add hydrogen atoms, supplement incomplete residues, delete alternate conformations, correct standardize atom order in amino acid and nonstandard names before docking assays.

The key amino acids of ACE2 were Lys31, Glu35, Asp38 and Lys353, while those of SARS-CoV-2 S-RBD were Tyr449, Leu455, Phe486, Tyr489, Gln493, Ser494, Gly496, Thr500, Asn501 and Tyr505. All amino acids residues around 5Å that centered on the inhibitor ligand were defined as the ACE2 enzyme active sites for molecular docking, according to the published literature [5,6,8]. After the protein and compounds were prepared, the CDOCKER module in Discovery Studio 3.0 was selected for molecular docking.

## Figures and Tables

**Table 1:** Kinetics/affinity evaluation of ACE2 and S-RBD with different inhibitors.

Compound	Target	*k_on_* (1/Ms)	*k_off_* (×10^−3^ 1/s)	K_D_ (×10^−6^ M)
02B05	ACE2	1989	3.345	1.736 ^a^
	S-RBD	2351	2.443	1.039 ^a^
02C15	S-RBD	2360	21.50	9.110 ^a^
02C19	ACE2	2344	52.05	22.21 ^a^
02J06	ACE2	2613	635.9	243.4 ^a^
	S-RBD	5123	328.9	64.20 ^a^
02M09	S-RBD	-	-	8.368 ^b^
03D12	ACE2	2764	9.480	3.429 ^a^
	S-RBD	2102	9.644	4.588 ^a^

^a^ Data were automatically fitted to the 1:1 binding model for kinetics. ^b^ Data were automatically fitted to the 1:1 binding model for steady state affinity.

## Data Availability

The data presented in this study are available on request from the corresponding author.

## Supplementary Materials

The following are available online, Figure S1: The results of immobilization of S-RBD on a CM5 sensor chip. Figure S2: The results of immobilization of ACE2 on a CM5 sensor chip. Figure S3: The sensorgram and structure of 02M09 identified from the affinity screening. Figure S4: The sensorgrams of SPR competition experiment. Figure S5: The results of cytotoxicity of compound 02B05. Figure S6: The results of pseudovirus entry assay of compound 03D12.
