# Supplementary material for: Blocking Effect of Demethylzeylasteral on the Interaction between Human ACE2 Protein and SARS-CoV-2 RBD Protein Discovered Using SPR Technology
Source: Molecules. 2020 Dec 24;26(1):57. doi: 10.3390/molecules26010057 (PMC7794844; doi:10.3390/molecules26010057)
Supplement: Supplementary file 1 [file molecules-26-00057-s001.pdf]

Supplementary Materials

Blocking Effect of Demethylzeylasteral on the Interaction between Human ACE2 Protein and SARS-CoV-2 RBD Protein Discovered Using SPR Technology

Zhi-Ling Zhu[1,†], Xiao-Dan Qiu[1,†], Shuo Wu[1,†], Yi-Tong Liu[1], Ting Zhao[1], Zhong-Hao Sun[1], Zhuo-Rong Li[1,*] and Guang-Zhi Shan[1,*]

Figure S1. The results of immobilization of S-RBD on CM5 sensor chip.

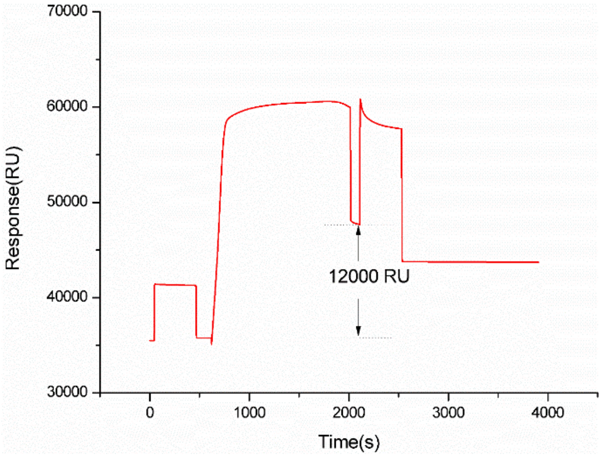

Figure S2. The results of immobilization of ACE2 on CM5 sensor chip

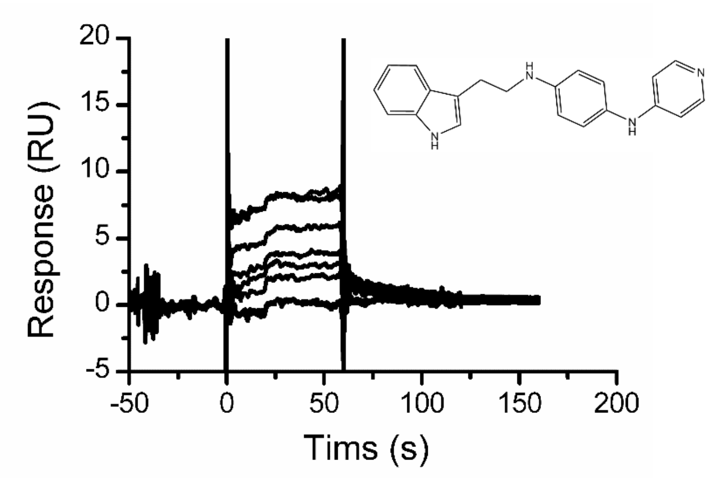

Figure S3. The sensorgram and structure of 02M09 identified from the affinity screening.

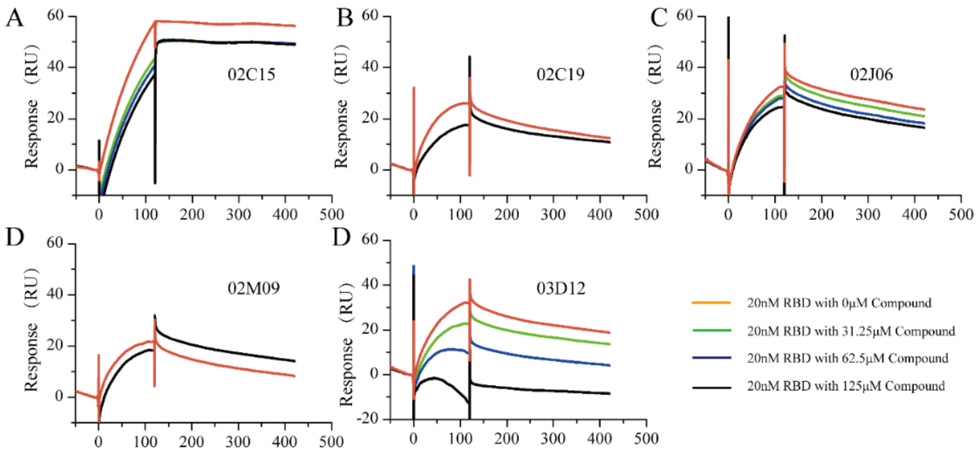

Figure S4. The sensorgrams of SPR competition experiment. The competitive effect of compound 02C15 and 02B05 were simultaneously determined using the same batch of protein; 02C19, 02J06, 02M09 and 03D12 were determined with the same batch of protein. Since the remaining amount of 02C19 and 02M09 is relatively small, only the inhibitory effect of 02C19 and 02M09 at the concentration of 125μM were measured.

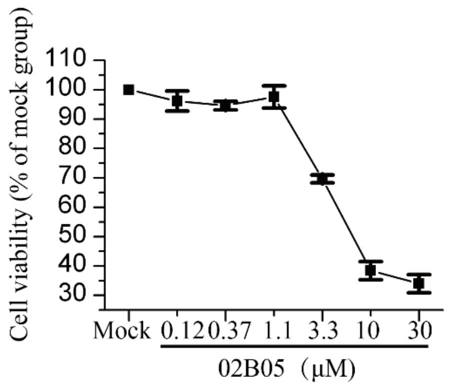

Figure S5. The results of cytotoxicity of compound 02B05 by a Cell Counting Kit (CCK) assay.

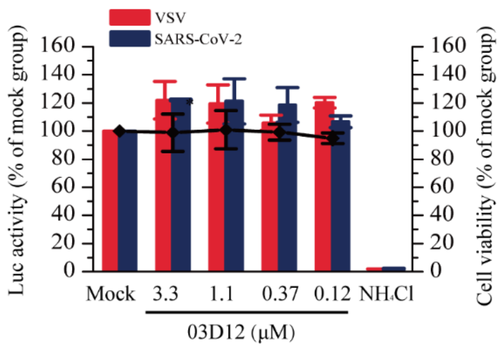

Figure S6. The results of pseudovirus entry assay of compound 03D12. The results revealed that no obvious inhibitory activity of 03D12 was observed at a concentration of 3.3 μM.
